# Physical rehabilitation modulates microRNAs involved in multiple sclerosis: a case report

**DOI:** 10.1002/ccr3.1100

**Published:** 2017-11-02

**Authors:** Annamaria Vallelunga, Carmine Berlingieri, Marco Ragusa, Michele Purrello, Maria Rosaria Stabile, Maria Consiglia Calabrese, Julio Cesar Morales‐Medina, Beniamino Palmieri, Tommaso Iannitti

**Affiliations:** ^1^ Department of Medicine and Surgery Centre for Neurodegenerative Diseases (CEMAND) University of Salerno Salerno Italy; ^2^ Fondazione “Ospedale San Camillo” IRCCS Venice Italy; ^3^ Department of BioMedical Sciences and BioTechnology Section of Biology and Genetics G Sichel BioMolecular, Genome and Complex Systems BioMedicine Unit (BMGS) University of Catania Catania Italy; ^4^ Physical Therapy and Rehabilitation. AOU S. Giovanni di Dio e Ruggi d'Aragona University of Salerno Salerno Italy; ^5^ Centro de Investigación en Reproducción Animal CINVESTAV Tlaxcala Mexico AP 62 CP 90000; ^6^ Department of General Surgery and Surgical Specialties Surgical Clinic University of Modena and Reggio Emilia Medical School Modena Italy; ^7^ KWS BioTest Marine View Office Park Portishead Somerset BS20 7AW United Kingdom

**Keywords:** Balance, gait, miRNAs, multiple sclerosis, neuromuscular taping, pain, physiotherapy

## Abstract

This study shows that neuromuscular taping improves gait, balance, pain and ability to walk and conduct daily activities in a multiple sclerosis patient. It is the first study to identify a panel of miRNAs modulated throughout rehabilitation using neuromuscular taping in a multiple sclerosis patient.

## Introduction

Multiple sclerosis (MS) is a neurodegenerative disease with symptoms including pain, coordination impairment, and muscle weakness 1. Rehabilitation can improve motor function and patients' quality of life (QOL). Neuromuscular taping (NMT) is a new elastic tape which improves muscular function, pain, and postural alignment, increases lymphatic and vascular flow, and strengthens weakened muscles 2. Furthermore, NMT increases leg muscle strength in patients affected by relapsing–remitting MS (RR‐MS) *versus* sham device 2. The concept of “rehabilomics” aims to study rehabilitation endophenotypes to discover the molecular substrates involved in rehabilitation, but no biomarker is available to determine rehabilitation efficacy. miRNAs are small noncoding RNAs responsible for post‐transcriptional gene regulation 3 and key regulators in MS 4, 6, 7, 9. In addition, they are modulated by exercise in healthy subjects 5, 8.

In this study, we determined NMT efficacy in a secondary‐progressive MS (SP‐MS) patient and investigated, for the first time, (1) whether circulating miRNAs are altered by NMT and (2) are predictors of successful rehabilitation therapy.

## Case Report

**Figure 1:**
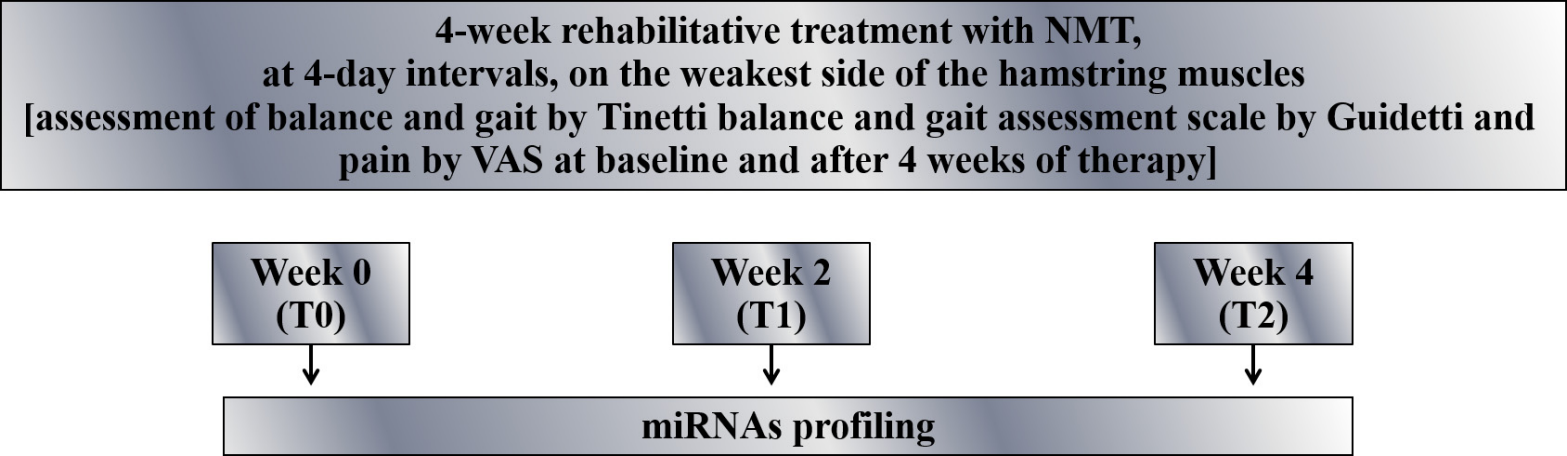
Diagram of rehabilitative treatment and serum miRNAs profiling. NMT, neuromuscular taping; VAS, visual analogue scale.

A 60‐year‐old Caucasian male patient, affected by SP‐MS for 23 years, presented to our hospital (Fondazione “Ospedale San Camillo” IRCCS, Venice, Italy) with an Expanded Disability Status Scale score equal to four. His first symptom was optic neuritis and was diagnosed at the age of 37. Besides spasticity‐ and ataxia‐related walking impairments, he displayed paraparesis, upper limb weakness, inability to make finest movements with his fingers, fatigue, dysarthria, liquid dysphagia, and bladder dysfunction without cognitive deficits. The patient signed the informed consent and was treated with NMT (the taping we used during the treatment was Cure Tape Beige, Aneid Italia S.r.l., Rome, Italy) for 4 weeks, at 4‐day intervals, on the weakest side of the hamstring muscles (Fig. 1). We used a 20‐cm‐long NMT which we subdivided into five fan‐like fringes and applied to create stand‐up folds. At baseline (T0) and after 4 weeks (T2), the patient was assessed using the Tinetti balance and gait assessment scale by Guidetti and the visual analogue scale (VAS) to assess pain (0 = no pain; 10 = maximum pain). The patient was asked to report any improvement in his lower limb strength and ability to walk for 10 meters and conduct daily activities (self‐assessed) following therapy. After patient's evaluation at T0, he underwent 1 h of regular physiotherapy daily for 5 days a week for a month, and NMT was applied twice a week for a total of 20 applications. All NMT applications followed David Blow's concept of decompression and lengthening of the skin. Correct NMT methodology is described as “decompression and dilation taping” that improves blood circulation and oxygenation to the treated area 10, 11. We applied NMT to treat the lumbar part of the *iliocostalis lumborum* and the *trapezius*,* deltoid*, and *gastrocnemius*, bilaterally, based on the patient's posture, balance, and pain. We hypothesized that rehabilitation combining NMT and physiotherapy would reduce pain and improve posture and balance, strengthen muscles, and improve QOL.

Serum samples were collected at T0, week 2 (T1), and week 4 (T2) and analyzed for miRNAs profiling using TaqMan Low Density Arrays 3 (Fig. 1). At T2, lower limb strength, ability to walk for 10 meters and conduct daily activities improved compared to baseline. Balance and gait improved from a total of 19 [balance=11; gait=8] at T0 to 7 [balance=4; gait=3] at T2. VAS pain score also improved from baseline [VAS (T0) = 8 cm; VAS (T2) = 2 cm]. During therapy, 53 miRNAs were deregulated. Twenty‐one miRNAs were upregulated, and 13 were downregulated at T1 and T2, if compared with T0. Sixteen miRNAs were downregulated at T1 and upregulated at T2 *versus* T0. Four miRNAs were upregulated at T1 and downregulated at T2 *versus* T0 (Table 1). In our miRNAs panel, we identified two miRNAs, mir‐140‐5p and mir‐642, upregulated throughout NMT treatment and previously shown to be involved in MS 4, 9, 14. We also identified four miRNAs (mir‐103, let‐7d, let‐7e, and mir223) modulated by exercise.

**Table 1:** miRNAs average fold change at T1 and T2 *versus* T0. miRNAs upregulated at T1 and T2 *versus* T0 are written in bold. miRNAs downregulated at T1 and T2 *versus* T0 are written in italics. miRNAs downregulated at T1 *versus* T0 and upregulated at T2 *versus* T0 are are written in italics and bold. miRNAs upregulated at T1 *versus* T0 and downregulated at T2 *versus* T0 are written in normal text

miRNAs	Fold change T1 *versus* T0	Fold change T2 *versus* T0
**MAMMu6**	3.02	6.18
**Hsa‐let‐7c**	2.72	3.88
**Hsa‐mir‐101**	1.03	2.54
**Hsa‐mir‐103**	1.74	2.23
**Hsa‐mir‐140‐5p**	1.50	2.78
**Hsa‐mir‐195**	2.30	2.05
**Hsa‐mir‐203**	3.29	2.57
**Hsa‐mir‐224**	6.54	13.31
**Hsa‐mir‐296‐5p**	3.94	1.85
**Hsa‐mir‐302c**	9.64	3.18
**Hsa‐mir‐320B**	2.86	1.76
**Hsa‐mir‐378**	1.04	2.14
**Hsa‐mir‐451**	2.17	1.13
**Hsa‐mir‐574‐3p**	5.68	14.70
**Hsa‐mir‐642**	4.31	1.07
**Hsa‐mir‐720**	2.05	1.77
**Hsa‐mir‐744**	2.06	4.11
**Hsa‐mir‐1233**	6.92	14.44
**Hsa‐mir‐1247**	4.29	1.13
**Hsa‐mir‐1260**	24.50	25.90
**Hsa‐mir‐1290**	1.48	6.25
Hsa‐mir‐144*	1.37	−39.59
Hsa‐mir‐194	9.60	−1.06
Hsa‐mir‐486‐3p	1.49	−5.74
Hsa‐mir‐491‐5p	1.69	−3.45
***Hsa‐mir‐125a‐5p***	−33.11	1.25
***Hsa‐mir‐128***	−4.40	1.01
***Hsa‐mir‐132***	−23.90	1.25
***Hsa‐mir‐133a***	−4.14	2.42
***Hsa‐mir‐134***	−6.62	1.13
***Hsa‐mir‐139‐5p***	−2.95	1.06
***Hsa‐mir‐146a***	−2.76	1.48
***Hsa‐mir‐150***	−2.92	1.09
Hsa‐mir‐223*	−2.74	1.17
***Hsa‐mir‐26a***	−3.10	1.05
***Hsa‐mir‐27a***	−19.96	1.12
***Hsa‐mir‐29a***	−3.44	2.65
***Hsa‐mir‐324‐5p***	−2.55	2.35
***Hsa‐mir‐339‐5p***	−7.06	1.21
***Hsa‐mir‐433***	−5.08	4.43
***Hsa‐mir‐605***	−1.09	2.62
*Hsa‐let‐7d*	−2.65	−1.44
*Hsa‐let‐7e*	−5.08	−1.14
*Hsa‐mir‐99b*	−4.84	−2.07
Hsa‐mir‐126*	−2.55	−2.15
*Hsa‐mir‐139‐3p*	−4.61	−1.51
*Hsa‐mir‐206*	−4.14	−4.28
*Hsa‐mir‐223*	−2.59	−1.01
*Hsa‐mir‐409‐3p*	−7.20	−1.09
*Hsa‐mir‐410*	−10.68	−4.27
Hsa‐mir‐425*	−4.35	−1.01
*Hsa‐mir‐495*	−15.77	−1.24
*Hsa‐mir‐942*	−8.38	−8.21
*Hsa‐mir‐1249*	−3.11	−2.76

## Discussion

We found that NMT improved gait, balance, pain and ability to walk and conduct daily activities in a MS patient. We hypothesise that this effect is due to the decompression and dilation created by the taping, which results in improved systemic blood circulation and oxygenation. This is in line with positive findings from a previous study by Camerota and coworkers who found an improvement in upper limb function after NMT in a female child with left hemiplegia due to cerebral palsy 12.

Wagner and coworkers introduced the concept of “Rehabilomics” as a field of study for rehabilitation endophenotypes using “Omics” science to discover the molecular substrates involved in rehabilitative processes and outcomes, personalising a biomolecular approach to rehabilitation care that is aimed for optimising individual recovery 13. We used this approach for the first time to quantify miRNAs in a patient during rehabilitative treatment.

We are the first to identify a panel of miRNAs modulated throughout rehabilitation in a MS patient. Evidence suggests that miRNAs are involved in MS pathogenesis and progression 4, 14 and are regulated by exercise 5, 8. We found 53 deregulated miRNAs throughout rehabilitation. Notably, we identified two miRNAs, mir‐140‐5p and mir‐642, upregulated throughout NMT treatment and previously shown to be involved in MS 4, 9. Guan et al. found that mir‐140‐5p was decreased in peripheral blood mononuclear cells (PBMCs) of patients affected by RR‐MS. In addition, they found an inverse correlation between expression of mir‐140‐5p and MS progression 9. mir‐642 was downregulated in MS patients' inactive brain lesions 4 and increased in whole blood from 19 natalizumab‐treated RR‐MS patients at 6‐ and 12‐month follow‐up *versus* baseline 14. This evidence supports our findings, suggesting that an improvement in MS symptoms is associated with an increase in miR‐140‐5p and mir‐642, while MS progression is associated with a reduction in miR‐140‐5p. We also found four miRNAs deregulated throughout rehabilitation, linked to MS and modulated by exercise 5, 6, 7, 8, 14. For example, mir‐103 was upregulated at T1 and T2 post‐NMT therapy *versus* baseline. Ingwersen and coworkers observed that mir‐103 was upregulated at 1‐year follow‐up in MS patients undergoing natalizumab treatment 7. In addition, Nielsen and coworkers observed an increase in mir‐103 after chronic training 5. We found a downregulation in let‐7d, let‐7e, and mir‐223 throughout NMT treatment. Let‐7d was upregulated in PBMCs from RR‐MS patients *versus* healthy controls 14 and downregulated in plasma from healthy trained men after 12‐week endurance training 5. Similarly, let‐7e was upregulated in the experimental autoimmune encephalomyelitis model 6 and downregulated in PBMCs of healthy young males after exercise 8. Finally, several studies found that miR‐223 was upregulated in MS regulatory T cells as well as plasma, blood cells, brain white matter, and active lesions, suggesting a role of mir‐223 in MS 4, 14. However, Nielsen and coworkers found mir‐223 downregulation in healthy men after 1 hour of acute aerobic exercise and endurance training 5. This supports our findings that NMT may improve MS symptoms modulating miRNAs. In conclusion, we found that NMT rehabilitative protocol improves MS modulating specific miRNAs and warranting further studies in a large cohort of patients to develop personalised rehabilitative protocols for MS.

## Ethics Approval and Consent to Participate

This study was approved by the institutional review board. The patient signed the informed consent.

## Consent to Publish

The patient signed the consent to publish.

## Authorship

AV, CB, and TI: performed experiments, wrote the manuscript, and created the table and figure. AV, CB, TI, MR, MP, MRS, MCC, BP, and JCMM: participated in acquisition of relevant literature and revised the manuscript critically for intellectual content. AV, BP, JCMM, and TI: participated in conception and design of the work. All authors: approved the final version of the manuscript.

## Conflict of Interests

The authors have no known conflict of interests to declare.
